# Integrated Piezoelectric AlN Thin Film with SU-8/PDMS Supporting Layer for Flexible Sensor Array

**DOI:** 10.3390/s20010315

**Published:** 2020-01-06

**Authors:** Hong Goo Yeo, Joontaek Jung, Minkyung Sim, Jae Eun Jang, Hongsoo Choi

**Affiliations:** 1 Department of Robotics Engineering, DGIST, Daegu 42988, Korea; hgyeo@dgist.ac.kr (H.G.Y.); Joontaek.JUNG@cea.fr (J.J.); 2 DGIST-ETH Microrobot research center, DGIST, Daegu 42988, Korea; 3 Department of Silicon Components, CEA-Leti, 38054 Grenoble, France; 4 Department of Information and Communication Engineering, DGIST, Daegu 42988, Korea; mksim@dgist.ac.kr (M.S.); jang1@dgist.ac.kr (J.E.J.)

**Keywords:** piezoelectric tactile sensor, aluminum nitride, thin film, flexible support layer

## Abstract

This research focuses on the development of a flexible tactile sensor array consisting of aluminum nitride (AlN) based on micro-electro-mechanical system (MEMS) technology. A total of 2304 tactile sensors were integrated into a small area of 2.5 × 2.5 cm^2^. Five hundred nm thick AlN film with strong c-axis texture was sputtered on Cr/Au/Cr (50/50/5 nm) layers as the sacrificial layer coated on a Si wafer. To achieve device flexibility, polydimethylsiloxane (PDMS) polymer and SU-8 photoresist layer were used as the supporting layers after etching away a release layer. Twenty-five mM (3-mercaptopropyl) trimethoxysilane (MPTMS) improves the adhesion between metal and polymers due to formation of a self-assembled monolayer (SAM) on the surface of the top electrode. The flexible tactile sensor has 8 × 8 channels and each channel has 36 sensor elements with nine SU-8 bump blocks. The tactile sensor array was demonstrated to be flexible by bending 90 degrees. The tactile sensor array was demonstrated to show clear spatial resolution through detecting the distinct electrical response of each channel under local mechanical stimulus.

## 1. Introduction

**Figure 1:**
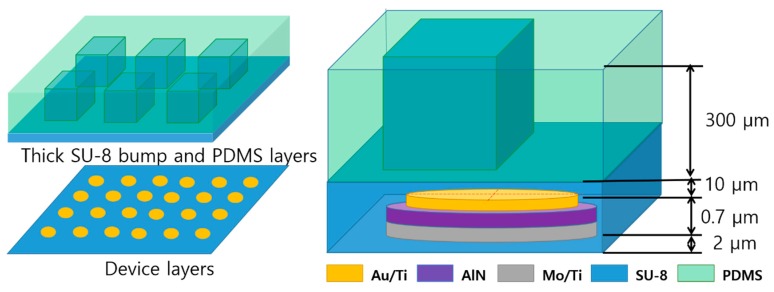
Schematic view of the flexible tactile sensor using SU-8 and polydimethylsiloxane (PDMS) polymers.

A range of information from the surrounding environment is acquired through sensory mechanisms such as taste, hearing, vision, and touch. Humans can sense the physical quantities of pain, softness, and shape and type of stresses through touch, known as tactile sensing [1,2]. With increasing demand for highly intelligent devices for teleoperation systems [3] or health monitoring [4,5], quantification of softness using electrical tactile sensors has become essential. Such applications require both accurate measurement of applied force, and the possibility of direct attachment onto the surface of objects such as organs or skin for wearable and implantable devices. Multiple approaches to artificial tactile sensors were investigated, using methods based on piezoresistive [6], capacitive [7], inductive [8], and piezoelectric [9] effects. Each of the sensing methodologies has advantages and disadvantages in tactile sensing [10]; however, among the sensing approaches, piezoelectric-based tactile sensors have particularly high sensitivity, a high dynamic range, and a high frequency response [11]. Nevertheless, piezoelectric tactile sensors are not able to detect static forces, because the impedance of a piezoelectric film is related to the decay of a developed charge under an applied force. In addition, the spatial resolution of piezoelectric tactile sensors using typical bulky piezoelectric ceramics is not as high as that of piezoresistive sensors due to the complexity of the fabrication process, which limits the size and structure of the device [3,10]. However, miniature tactile sensors based on piezoelectric thin films may lead to improvements in the spatial resolution of tactile sensors with low operating power consumption. Piezoelectric tactile sensors can be successfully down-scaled through advanced micro-electro-mechanical systems (MEMS) technology [12,13]. A high piezoelectric constant and a low dielectric constant are essential to achieve high sensitivity for piezoelectric MEMS-based devices [14]. Among various piezoelectric thin films, such as aluminum nitride (AlN) [15], ZnO [16], BaTiO_3_ [17], lead zirconate titanate (Pb(Zr,Ti)O_3_, PZT) [18] and polyvinylidenefluoride (PVDF) [19], PZT is the most popular due to its high piezoelectric constant, high energy density, and large electrical-mechanical coupling coefficients [12,20]. However, the pressure/touch sensitivity of thin film PZT with regard to voltage response is slightly smaller than that of AlN [21] because of a large dielectric constant—two orders of magnitude higher than that of AlN. Moreover, PZT film should be annealed at a high temperature (>600 °C) for crystallization of the perovskite structure, and contamination problems may be encountered [22] in a standard complementary metal oxide semiconductor (CMOS) cleanroom [23]. PVDF is regarded as a good candidate for fabrication of flexible tactile sensors, because it presents high sensing sensitivity due to its low dielectric constant of ~50, light weight, ease of fabrication and excellent flexibility [24]. Using these characteristics of PVDF film, some studies presented PVDF film-based tactile sensors [9,25,26]. However, PVDF film provides only a limited working temperature due to its low glass transition temperature of −40 °C [27]. Based on the transition and Curie temperatures, the working temperature of PVDF film is limited to between −20 and 70 °C [28,29]. Additionally, in terms of tactile sensing for multiple stimuli, undesirable coupling of soft piezoelectric polymers between neighbor sensors can occur through high mechanical transmission, and may be a critical issue for tactile sensor arrays with respect to detecting multiple stimuli [30]. Unlike PVDF film, AlN thin films show a wide working temperature range, from −196 to 1150 °C, with a high electromechanical coupling coefficient due to the low dielectric permittivity and relevant piezoelectric coefficient [29]. In particular, in recent decades, many researchers have become interested in growth of AlN on flexible polymeric and metal foil substrates to enlarge the field of applications of fabricated AlN sensors [31,32,33,34]. However, it is still rarely reported for flexible MEMS tactile sensor array using AlN thin film [35]. In most cases, the reported flexible tactile sensors are about polymer piezoelectric materials such as PVDF. Here we propose a MEMS fabrication process for an AlN-based tactile sensor array with poly (dimenthylsiloxane) (PDMS) and an SU-8 bump structure, which serves as a contact force (including normal and shear force) for translation into four piezoelectric transducers (Figure 1). The 500 nm thick AlN thin film was grown by sputtering at room temperature. After releasing the tactile sensor from the rigid silicon (Si) substrate with a chrome sacrificial layer, the tactile sensor was placed on a 2 μm SU-8 thin layer. A square-shaped SU-8 bump structure was placed in the middle of four AlN-based tactile sensors covering the four corners of the sensors to enhance the pressure sensitivity [35].

## 2. Fabrication Process of the Tactile Sensor Array

**Figure 2:**
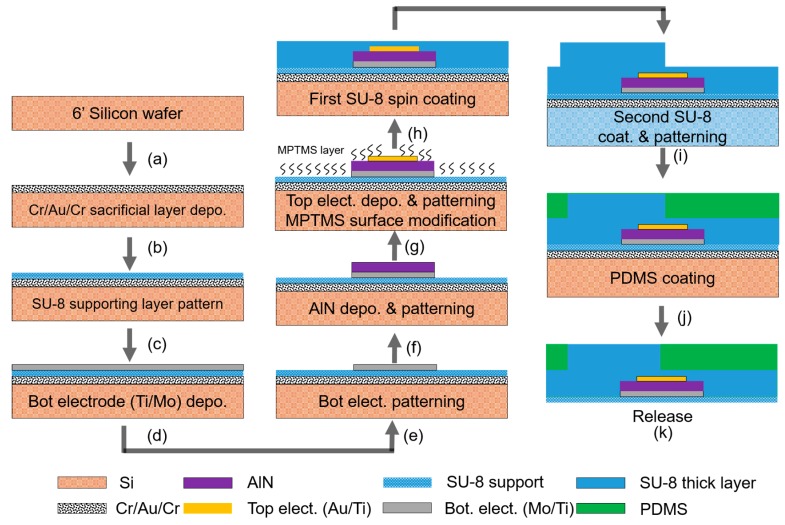
(**a**–**k**) Fabrication process of flexible tactile sensor.

The fabrication process for the proposed flexible tactile sensor array started with the 6-inch <100> P-type Si wafer (thickness of 675 µm) shown in Figure 2a. To release the device from the Si substrate using chrome etchant, Cr/Au/Cr (5/50/50 nm) sacrificial layers were deposited on the Si wafer at room temperature using an e-beam evaporator (SRN-200; Sorona, Anseong-si, South Korea) (Figure 2b). Following this, the supporting SU-8 2002 (Kayaku Advanced Material, Westborough, MA, USA) photoresist layer was spun and patterned using a photolithography process (Figure 2c). The supporting layer was then hard-baked at 150 °C for 10 min to avoid stripping of the device layers during the lift-off process for the bottom electrode [36].

A 100/10 nm thick molybdenum/titanium (Mo/Ti) layer was sputtered (SRN-110; Sorona, Anseong-si, South Korea) as the bottom electrode at room temperature and patterned using a lift-off process with AZ 5214 photoresist (Figure 2d,e). The lift-off process was used to obtain patterned AlN and a top Au/Ti (100/10 nm) electrode, using the same photoresist and fabrication method as for the bottom electrode (Figure 2f,g). The AlN layer was grown using reactive sputtering (SRN-120; Sorona, Anseong-si, South Korea) with an Al target (99.9999%, 10 inch) at room temperature under 2.5 mTorr with a gas mixture of N_2_ (40 sccm) and Ar (10 sccm). The distance between target and substrate is 50 mm. An AlN film thickness of around 500 nm was achieved for 3521 s in this deposition conditions. After patterning the device layer, its surface was modified to improve adhesion with the SU-8 thick layer and bumps. To enhance the bonding strength between the top electrode and the SU-8 layer, a self-assembled monolayer (SAM) was established using (3-mercaptopropyl) trimethoxysilane (MPTMS), which served as an adhesive layer [37,38,39,40]. For the MPTMS solution, 46 μL MPTMS (95% purity; Sigma-Aldrich, St. Louis, MO, USA) was added to 10 mL methanol (25 mM MPTMS solution). The substrate was immersed in the MPTMS solution for 1 h and dried in air for 10 min. After surface modification, the 10 μm thick first SU-8 2010 layer was formed by spin-coating (Figure 2h). The second 300 μm-thick SU-8 2100 was spun on the passive layer of the first SU-8 2010 and patterned to form SU-8 bumps (Figure 2i). The PDMS base and agent (Sylgard 184; Dow Corning, Midland, MI, USA) were mixed at a ratio of 10:1. The mixed PDMS solution was poured on the device and then a manual doctor blade method using a square-shaped glass slab was applied to fill the gaps between the 300 μm-thick SU-8 bumps (Figure 2j). The PDMS layer was thermally cured at 60 °C for 4 h. After fabrication of the device, a 6-inch Si wafer was diced using a sawing blade for exposure of the etchant through the edge of each device, to improve the etching rate of the sacrificial layers. The 6-inch Si wafer was immersed in the chrome etchant (CR-7; KGM Chemicals, Fort Worth, TX, USA) for 6 h to release the tactile sensor array from the Si substrate (Figure 2k). The optimal timing for full release of large-scale devices requires attention. It is essential to minimize etch-induced damage to the AlN layer.

## 3. Tactile Sensor Fabrication and Experiment Results

### 3.1. Fabricated Tactile Sensor Array

**Figure 3:**
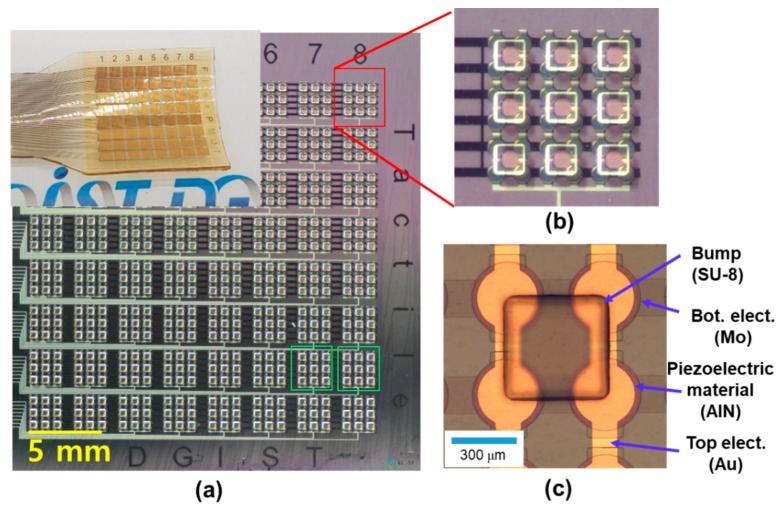
Optical view of (**a**) tactile sensor array (insert: fabricated flexible tactile sensor array after releasing from substrate) and (**b**,**c**) magnified view of sensor elements and SU-8 bumps.

The flexible tactile sensor array was successfully fabricated without obvious cracking of the metal layer (as shown in Figure 3). The tactile sensor array has 8 × 8 channels with 3 mm center pitch (the distance between channels) to read electrical signals. The output signal of each channel is collected from 36 tactile sensing elements. Few tactile sensor arrays with a large number of elements were reported; here, a total of 2304 active elements were fabricated over an area of 2.5 × 2.5 cm. The membrane diameter and center to center pitch of the tactile sensor element are 300 and 400 μm, respectively. Not only the gaps between SU-8 bumps but also the 500 μm gaps between neighboring channels (green boxes) were filled with PDMS polymers for flexibility. Based on the flexible capabilities of the PDMS, an increase in the gap could enhance the flexibility [41]. The 10 μm thick first SU-8 membrane layer was used to shift the neutral axis out of the piezoelectric material (Figure S1 in the Supplementary Materials) [42]. The 300 μm-thick SU-8 bump has the area of 400 × 400 μm^2^ and its four corners lie on the center of each piezoelectric element. The SU-8 bump structure would be enough to transfer force or generate torque in piezoelectric element due to relatively big size and large height of the bump [35]. The PDMS layer with low Young’s modulus surrounding each SU-8 bump enhances flexibility and mechanical reliability [43]. 

**Figure 4:**
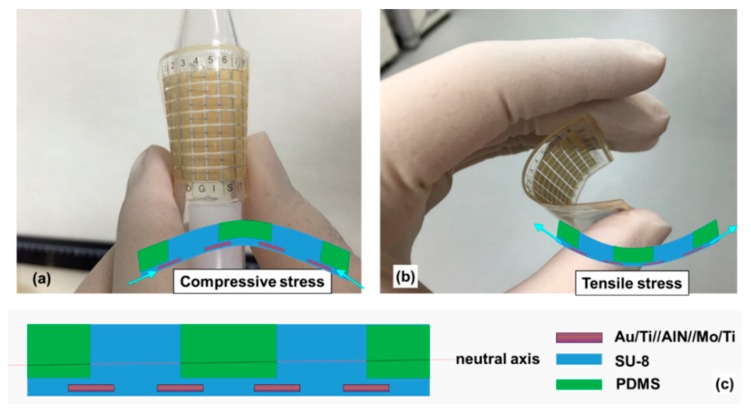
Bending demonstration of tactile sensor under (**a**) compressive bending and (**b**) tensile bending of flexible tactile sensor. (**c**) Schematic of flexible tactile sensor structure.

Figure 3b,c show a magnified view of the tactile sensor. The fabricated tactile sensor array mainly comprises SU-8 and PDMS that many research teams are used to encapsulate or make a microstructure on bio-devices [44,45,46]. By virtue of the 2 μm SU-8 supporting layer, the metal layer of the tactile sensor array shows good consistency even after release from the Si substrate. To demonstrate flexibility, Figure 4a,b show the tactile sensor bent by two fingers. Figure 4c is a schematic showing the flexible tactile sensor (a multilayer piezoelectric AlN) in the neutral plane, where no strain is encountered (Equation (S1) in the Supplementary Materials). When the device was bent into a convex circular arc, the inner surface (below the neutral plane) was compressed; however, it was expanded if a concave arc formed. Thus, piezoelectric thin layers, such as AlN, are subjected to compressive stress (Figure 4a) [47]. When the applied forces of compressive bending (i.e., piezoelectric AlN elements (red bars) were contracted along the in-plane direction in Figure 4a) and tensile bending (i.e., piezoelectric AlN elements (red bars) were elongated along the in-plane direction in Figure 4b) were larger than 90°, the flexible tactile sensor did not exhibit any mechanical fracture; hence, the fabricated tactile sensor array using 500 nm thick AlN film with polymer structures shows excellent flexibility without damage to the device. 

**Figure 5:**
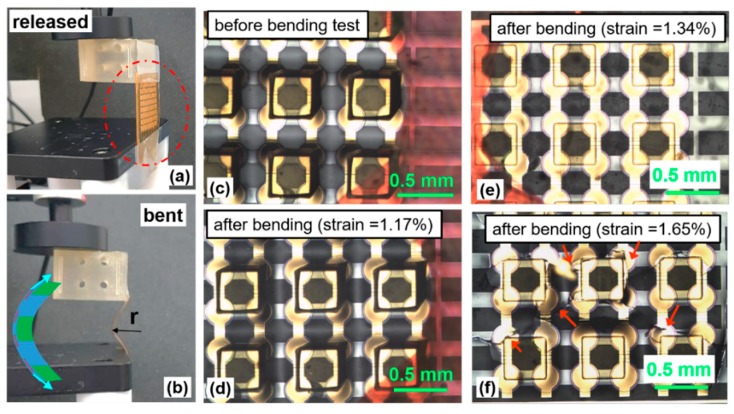
Periodic bending test. The original (**a**) and bent (**b**) sensor. Top down optical microscopic images of the flexible tactile sensor (**c**) before and (**d**–**f**) after 250 cycles of bending/unbending at various strain levels. We assessed mechanical durability and flexibility limitations (Strain limitation is below 1.65%).

Additionally, the mechanical durability of the flexible tactile sensor was characterized after periodic bending and release using a linear motor. A vertically mounted flexible tactile sensor (see red dot circle in Figure 5a) between stage and load block was bent 1000 times under various radii of curvature resulting from vertical displacement (i.e., different strains, which is calculated by Equation (S2) were imposed). Programmed deformation of a flexible tactile sensor by a linear motor induces tensile stress in the piezoelectric layer (red bar in Figure 5b). Figure 5d shows a top down view of the sensor prior to the bending test. No cracking or delamination was noted after 1000 bending/unbending cycles at a radius of curvature of 10.6 mm (corresponding to a strain of 1.17% at 0.8 Hz; Figure 5d) [48]. To further explore the mechanical limitations, the radius of curvature used in later tests was reduced, increasing the strain to 1.65% resulting in 10 mm vertical displacement. Significant cracks developed after 250 bending cycles above 1.65% of strain level. The cracks in the piezoelectric AlN layer sensor are indicated by red arrows in Figure 5f. This flexibility and mechanical durability are expected to be useful in a range of applications, such as health care, human-machine interfaces such as robotics, invasive surgical tools, and textiles and clothing [10].

### 3.2. Characterization of the Tactile Sensor Array

**Figure 6:**
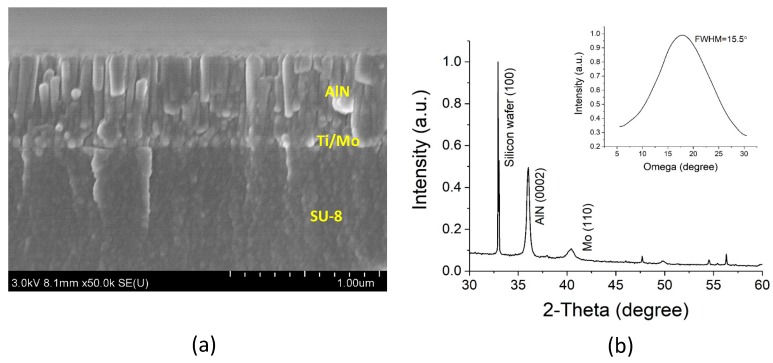
(**a**) Cross-sectional scanning electron microscopy (SEM) image of the aluminum nitride/molybdenum/titanium (AlN/Mo/Ti) layer on the substrate. (**b**) X-ray diffraction pattern and rocking curve (inset) of the film.

Figure 6a shows a cross-sectional view of the AlN/Mo/Ti/SU-8 film (500/100/10/2000 nm) on the Si substrate. A smooth surface and columnar grains (~70 nm) perpendicular to the surface of the SU-8 supporting layer were observed on the AlN thin film. Figure 6b shows the X-ray diffraction (XRD) pattern and rocking curve of the stacking layers of the AlN/Ti/Mo/SU-8 film on the Si substrate before release. The main peak observed is the (0002) hexagonal AlN peak at 2θ ≈ 36° and the full width half maximum (FWHM) of the XRD rocking curve is 15.5°. Elemental distributions across the release layers were confirmed by Energy-dispersive X-ray spectroscopy (EDS) on the dual beam focused ion beam system (B 5000; Hitachi, Tokyo, Japan) as shown in Figure S2. Laser Doppler Vibrometry (LDV; Polytec GmbH, Waldbronn, Germany) was used to measure the out-of-plane displacement induced by an AC electric field. A converse piezoelectric effect was evident. However, LDV cannot directly measure the piezoelectric coefficient because a structure-induced clamping effect deflects the substrate [49]. Unfortunately, the coefficient of a micromachined piezoelectric thin film is difficult to measure, unlike the case for bulk material or a simple, continuous piezoelectric thin film on a Si wafer. Complex composite structures (several layers on a substrate) lack the standard boundary conditions required for quantification of the piezoelectric coefficient [12,50]. 

**Figure 7:**
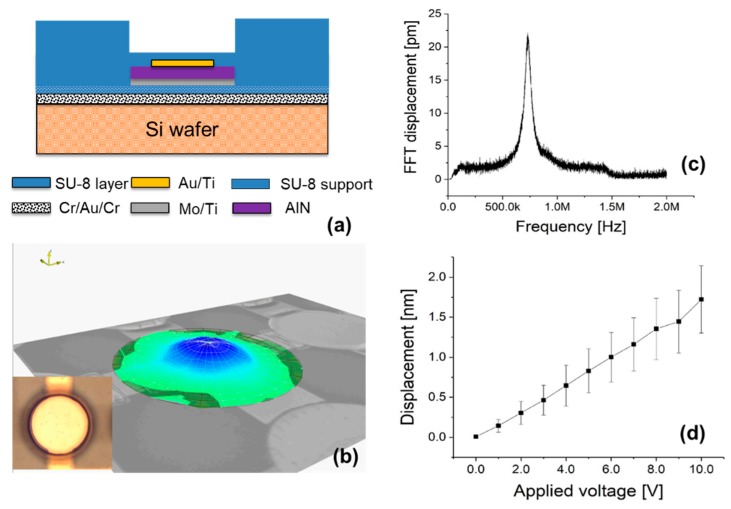
(**a**) Schematic showing the cross-section of an AlN element on an Si wafer without SU-8 bumps, used to measure displacement. (**b**) The displacement amplitude profile of the piezoelectric AlN element at the resonance frequency (inset: Top down optical image of the AlN element on the Si wafer). (**c**) Frequency response measurements of displacement at the center of the membrane. (**d**) Displacement as a function of the electrical voltage applied at the resonance frequency (740 kHz).

Basically, the out-of-plane deflection of the piezoelectric layer is strongly influenced by substrate and structure. Displacement profiles from the surface of the fully clamped AlN film on the SU-8-coated Si substrate in Figure 7b were captured from the animation recorded by LDV (Video S1, in Supplementary Materials) and shows the shape of the flexure mode (0, 1). To minimize any clamping effect imposed by the structure, which could reduce the converse piezoelectric effect and enhance surface reflectivity, the AlN membrane of Figure 7a was not covered with SU-8 bumps (Figure 7d shows the measurements obtained using the LDV). Figure 7c shows the dynamic displacement amplitude of the 300 μm-diameter AlN membrane with frequency induced by applying a periodic chirp voltage signal (10 V) at zero bias voltage. The peak displacement of the piezoelectric AlN element is shown at its resonance frequency of 740 kHz. The average of five individual piezoelectric AlN element displacement amplitudes as a function of sine wave voltage is plotted in Figure 7d. Since all vertical displacements per volt are constant over the range of applied voltage, the electric field-induced displacement represents a typical piezoelectric behavior, such as actuation [50]. Based on these results, we can conclude that crystallized piezoelectric AlN thin films were grown effectively on SU-8 at room temperature using the reactive sputtering technique. 

**Figure 8:**
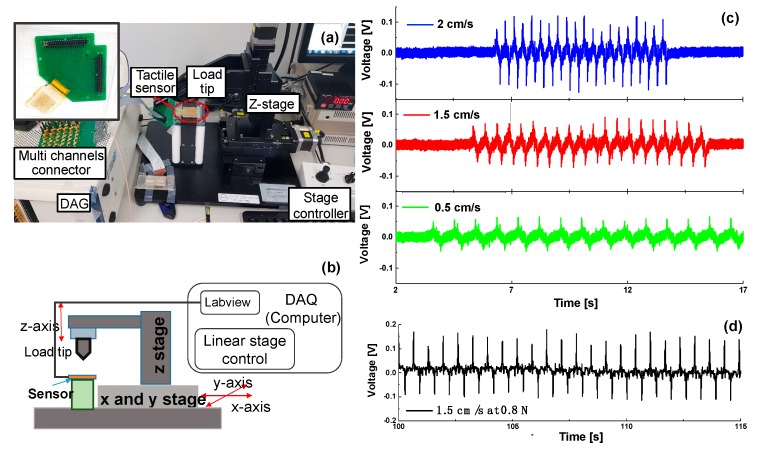
(**a**) Picture and (**b**) block diagram of the experimental setup with data acquisition (DAQ) equipment. (**c**) Voltage signal at different load speeds under force (0.2 N). (**d**) Voltage signal at 1.5 cm/sec of load speed under 0.8 N force load.

A force measurement system using a data acquisition (DAQ) system (National Instruments, Austin, TX, USA) and a linear stage was built to evaluate the sensing properties of the tactile sensor. The probe tip can control the position of the load. The DAQ is used to obtain piezoelectric response from sensor and control the linear stages and signals processing steps simultaneously (Figure 8b). Details on the setup for pressure measurements are provided elsewhere [51]. As shown in Figure S3a, the sensor array electrodes, with a pitch of 300 µm with respect to the 300-µm-wide electrode terminal, were connected to the printed circuit board (PCB) and placed on a stage. To make this connection, the sensor electrode and electrode pad (Figure S4) on PCB were aligned using an anisotropic conductive film (ACF) bonder (GZC-YPJ09) and bonded together with the z-axis (i.e., the thickness direction) conductive adhesive transfer tape (3M ECATT 9703), as shown in Figure S4 of the Supplementary Materials. The tape allows for electrical conduction throughout the adhesive and mechanically bonds the flexible tactile sensor to the PCB, without the need for thermal curing. 

The open-circuit output voltage of the tactile sensor to a force of 0.2 N, applied using a plastic probe tip 1.5 mm in diameter along the z-axis, is shown in Figure 8a,b. The vertical load and unload (z-axis) speeds were 0.5, 1.5, or 2 cm/s (1.2, 1.8, and 2.6 Hz respectively). Figure 8c shows that the peak voltages were 56, 72, and 97 mV in response to the various stimuli frequencies when a loading force of 0.2 N was applied 20 times to the single channel. As the rate of motion increased under the same applied force (0.2 N), the peak output voltage increased significantly. Below 0.2 N, however, the output signal was not apparently detectable at low loading speed. The sensitivity of a piezoelectric tactile sensor depends strongly on the dynamic excitation rate. When the stroke is completed, the voltage instantly returns to the initial level within the defined noise range, which is not the case for an electrical conductance-based tactile sensor [52,53]. This confirmed that the piezoelectric tactile sensor is suitable to detect a dynamic force. As shown in Figure 8d, the peak voltage of 142 mV, which is almost 100% higher than the applied loading force of 0.2 N (the 72 mV of peak voltage in Figure 8c), was generated by a load force of 0.8 N delivered at 1.8 Hz.

**Figure 9:**
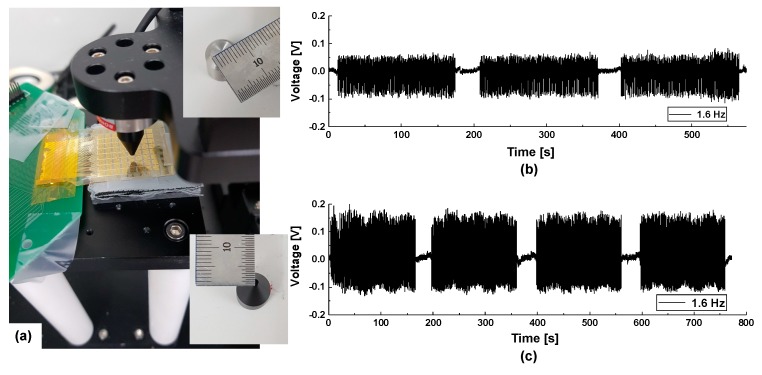
(**a**) Picture of experimental setup with probes (diameter of 7 mm and 1.5 mm) with device on stage. (**b**) Voltage response at the frequency of 1.6 Hz under 0.8 N force load with probe tip 1.5 mm in diameter (**c**) Voltage response at the frequency of 1.6 Hz under 1.2 N force load with probe tip 7 mm in diameter.

To determine the reliability of the AlN-based tactile sensor, the force of the 0.8 N and 1.2 N at the frequency of 1.6 Hz was applied to the sensor using the probe tips 1.5 mm and 7 mm in diameter respectively. As shown in Figure 9c, the voltage response is not shown any noticeable degradation under 1.2 N force load during pressing of 950 cycles. Additionally, to confirm the functionality of piezoelectric flexible tactile sensor detecting pressure on curvature surface and the robustness, the AlN based on the piezoelectric tactile sensor demonstrated a 250-cycle touch test with probe tip 7 mm in diameter at 1.1 Hz under tensile stress through the sensor attached to metal rod (radius of curvature = 6.5 mm, induced strain of 1.9%) (see Figure S5).

**Figure 10:**
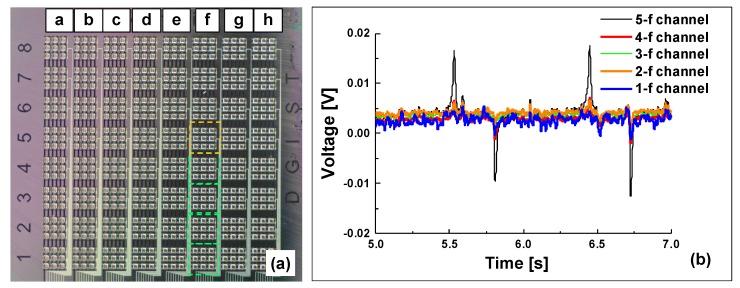
Mechanical stimulation of the sensor and measurements in the activated channel. (**a**) In top down view of sensor array, the orange circle indicates the stimulation area where probe tip is loaded 7 mm diameter of probe tip (**b**) Measurement in the channels (green squares) with applied mechanical stimulation region (orange circle).

We explored crosstalk effects in the flexible tactile sensor array by measuring the voltage responses of the neighboring channels. As the probe tip (contact area, 3.8 × 10^−5^ m^2^) driven by the linear motor applied a constant pressure to the 5-f channel (orange circle) at 1.1 Hz, we measured the outputs of sensing elements adjacent to that channel simultaneously (i.e., the 4-f, 3-f, 2-f, and 1-f channels; green squares in Figure 10). On loading using the stainless steel tip, a peak output voltage of 0.02 V was generated in the channel (5-f) where the load was applied, as shown in Figure 10b. Although the nearest neighboring channel (4-f) exhibited a weak peak signal (33% of that from 5-f) because it was partially pressurized by the large probe tip, the peak voltage from channel 5-f clearly differed from the signals of adjacent channels that were not under pressure. Figure S6 shows the response property of the tactile sensor under an artificial input through tapping using a cylindrical silicone rubber rod (diameter: 5 mm, length: 3 cm). No external resistance load was used and the output signal from the tactile sensor was directly measured using an oscilloscope at 1 MΩ input resistance (DSOX2004A; Agilent, Santa Clara, CA, USA). Video S2 (Supporting Information) shows the performance of the flexible tactile sensor array when measuring the electrical output from one channel. To evaluate the electrical interference between channels, a silicone rubber rod was pressed on the bump of a tactile channel (5-e). The peak signal voltage (red arrows) of 5-e sensing channel is apparently observable under tapping in Figure S6b. As shown in Figure S6d, the output signal oscillated between −0.25~0.06 V, which falls within the range of electrical noise, while tapping in the area (6-g) deviating from the channel connected to the oscilloscope. Negligible crosstalk could be attributed to the low modulus of the PDMS within channels.

## 4. Conclusions

A fabrication process for a tactile sensor array based on an AlN thin film was developed successfully on a SU-8 supporting layer with bumps that served to translate the contact force. The flexible tactile sensor array had 2304 tactile elements in an area of 2.5 × 2.5 cm. The MPTMS surface treatment is essential for improving the adhesion strength between gold and polymers (SU-8 photoresist and PDMS). The fabricated tactile sensor shows good flexibility and mechanical integrity on the curved structure. A c-axis (0002)-oriented AlN thin film with a FWHM of 15.5° was deposited on the polymer layer using rf-magnetron reactive sputtering. This demonstrates the capability of the tactile sensor for detection of dynamic stimuli in real time without significant interference between channels. In particular, we demonstrated that the piezoelectric flexible tactile sensor using an AlN layer with bumps is suitable for detection of dynamic motion/touch with a sensitive response at small force (0.2 N).

In addition, this microfabrication technique for a flexible tactile device with high sensor density could represent an advanced fabrication method for numerous applications, ranging from ultrasound transducers to energy harvesting, by adding functionality such as flexibility and bio-compatibility.

## Supplementary Materials

The following are available online at https://www.mdpi.com/1424-8220/20/1/315/s1, Video S1: Displacement of amplitude profile of the piezoelectric AlN element, Video S2: Mechanical stimulation of the sensor.
