# Supplementary material for: Integrated Piezoelectric AlN Thin Film with SU-8/PDMS Supporting Layer for Flexible Sensor Array
Source: Sensors (Basel). 2020 Jan 6;20(1):315. doi: 10.3390/s20010315 (PMC6982933; doi:10.3390/s20010315)
Supplement: Supplementary file 1 [file sensors-20-00315-s001.zip › Supplementary Materials_final.docx]

Supplementary Materials

Integrated Piezoelectric AlN Thin Film with SU-8/PDMS Supporting Layer for Flexible Sensor Array

Hong Goo Yeo ^1,2^, Joontaek Jung ^1,2,3^ , MinKyung Sim ^4^, Jae Eun Jang ^4^ and Hongsoo Choi ^1,2,^*

^1^ Department of Robotics Engineering, DGIST, Daegu, 42988, Korea

^2^ DGIST-ETH Microrobot research center, DGIST, Daegu 42988, Republic of Korea

hgyeo@dgist.ac.kr (H.G.Y.)

^3^ Department of Silicon Components, CEA-Leti, Grenoble, 38054, France

Joontaek.JUNG@cea.fr (J.J.)

^4^ Department of Information and Communication Engineering, DGIST, Daegu, 42988, Korea

mksim@dgist.ac.kr (M.S.); jang1@dgist.ac.kr (J.E.J.)

***** Correspondence: mems@dgist.ac.kr; Tel.: +82-53-785-6212 (H.C.)

**Calculation for the neutral axis of tactile sensor based on AlN**

The mechanical neutral axial (Z) can be calculated as follows:

$Z=\frac{\sum_{i=1}^{n} Y_{i}h_{i}z_{i}}{\sum_{i=1}^{n} Y_{i}h_{i}}$ (S1)

Where n is the total number of layers, z_i_ is the height of centroid of the layer i, hi is the thickness of the layer i and Y_i_ is Young’s modulus of the layer i. For AlN based tactile sensor, the Young’s modulus and thickness of each layers are: Y_AlN_ = 330 GPa, Y_PDMS_ = 0.87 GPa, Y_SU-8_ = 4.02 GPa, Y_SU-8_ = 4.02 GPa, Y_Au_= 78 GPa, Y_Ti_= 116 GPa, Y_Mo_= 329 GPa,


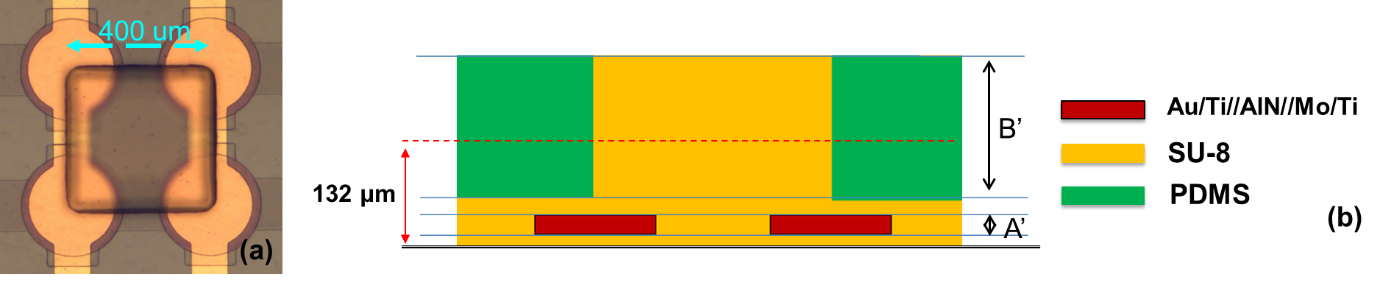


**Figure S1.** (a) Top-down view of four pMUT elements and one SU-8 bump. (b) Schematic showing the cross section of piezoelectric layer with electrodes (dark red), SU-8 layer (yellow) and PDMS (green) with neutral plane (red dot line).

To be simplified Young modulus of A’ layer, we consider the volume fractions of piezoelectric layer with electrodes and SU-8 in B’ layer which are 75% and 25% respectively. For The volume fraction of SU-8 and PDMS in B’ layer is 50% and 50% respectively. One channel consists of four membranes with one SU-8 bump. Strain on the plane of the tactile sensor can be expressed by Eq. (S2)

Strain (ε) = δ/r (S2)

Where δ is the distance between the neutral plane and the mid plane position of the AlN layer and r is the bending radius of the tactile device. r is bending radius. Total thickness of flexible tactile sensor is around 319 µm.


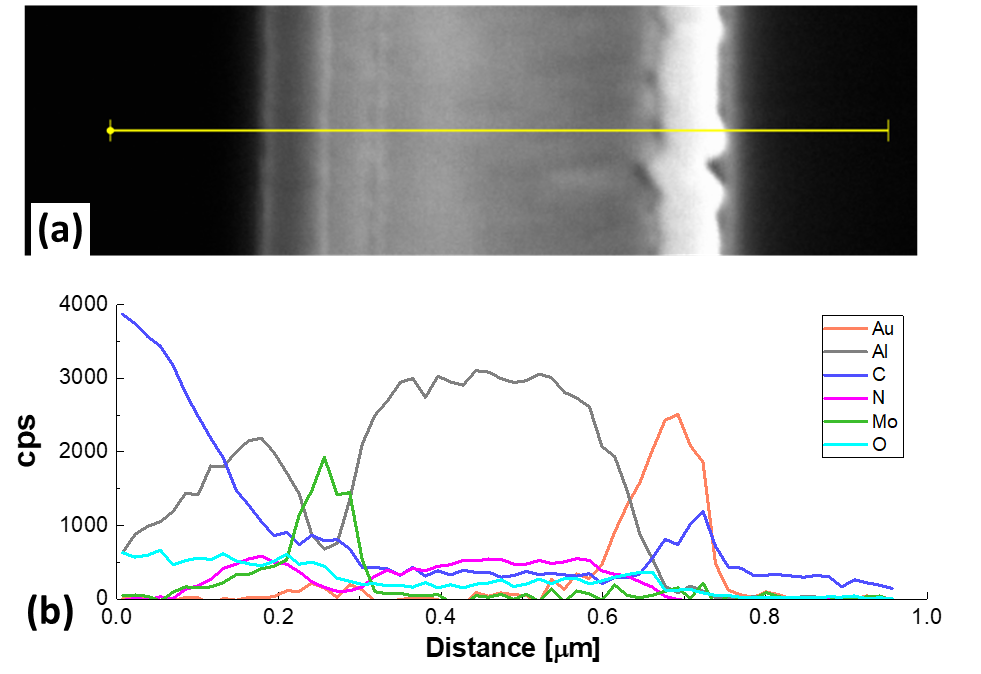


**Figure S2.** (a) Cross-sectional microstructure image and (b) corresponding EDS elemental line of the stacking layer.


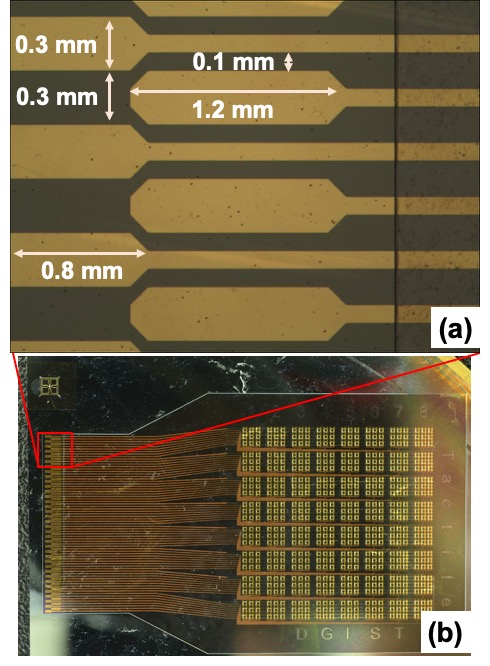


**Figure S3.** (a) Magnified top view of electrode terminals for connection with PCB. (b) Top view of tactile sensor array electrode configuration before releasing.


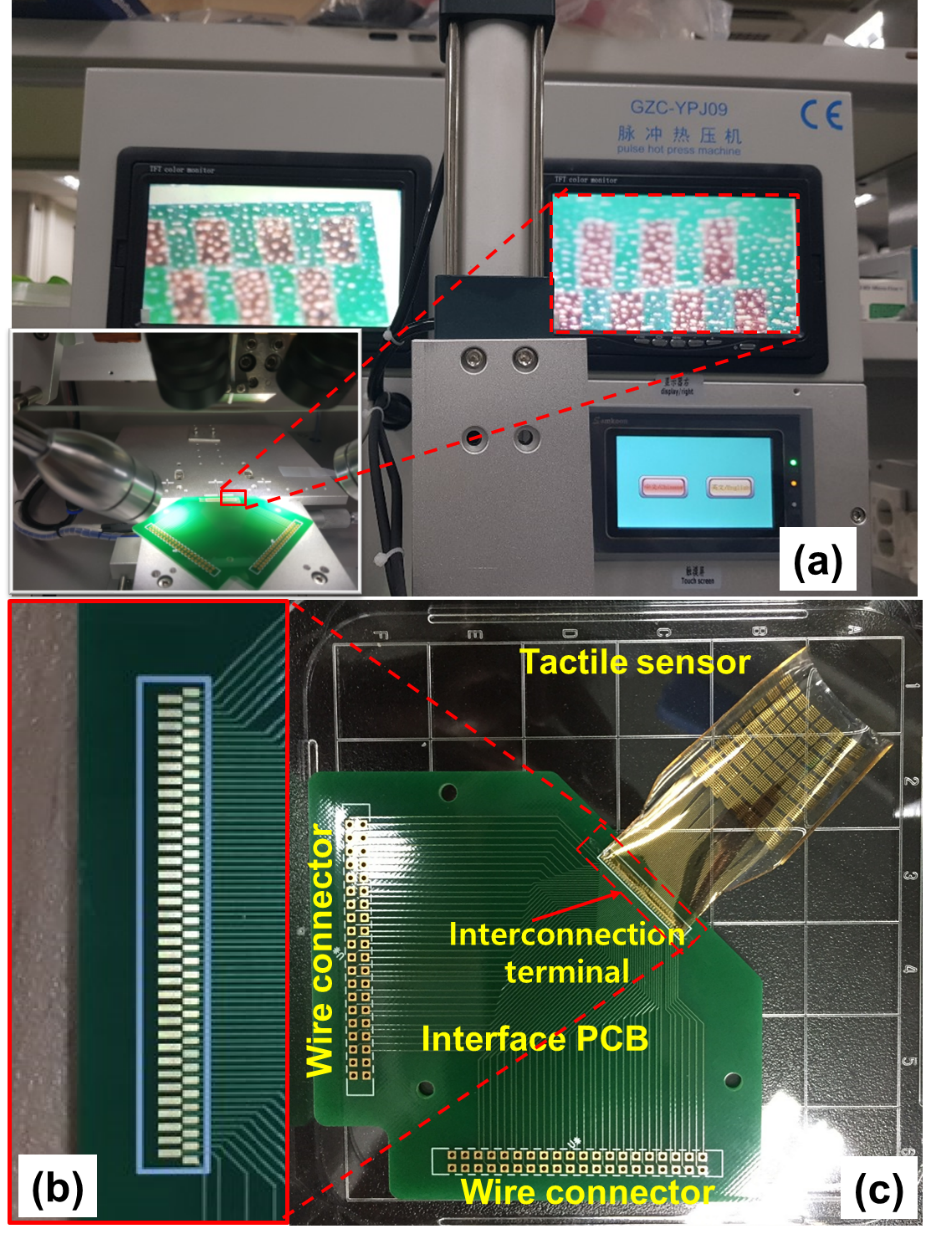


**Figure S4.** (a) ACF bonder(GZC-YPJ09) to align and bond PCB and the sensor electrodes with anisotropy conductive tape (insert: PCB with conductive adhesive transfer tape placed on the stage). (b) Image of electrode pad on PCB before bonding and (c) completed flexible tactile sensor with interconnection terminal.


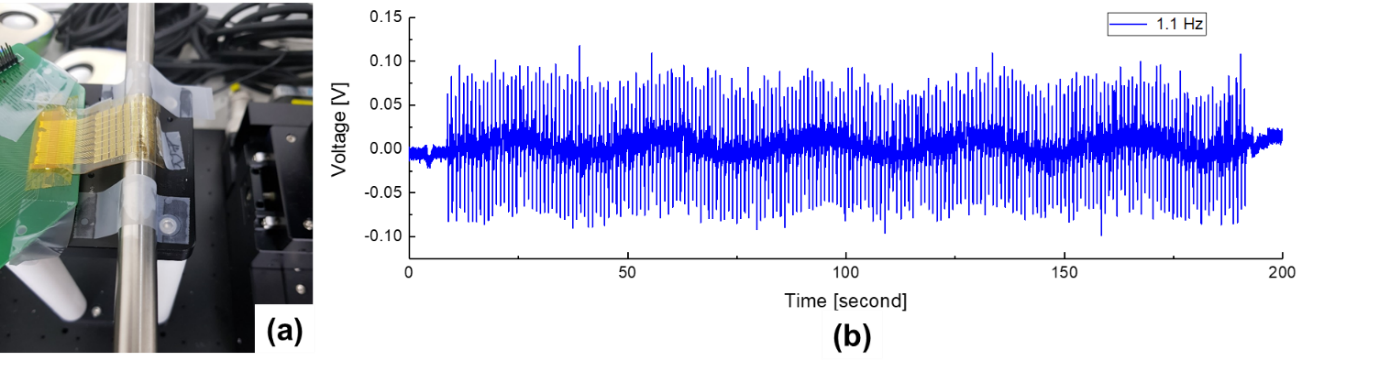


**Figure S5.** (a) Image of tactile sensor placed on metal rod for measurement of mechanical stability under curvature. (b) Voltage response at 1.1 Hz of load speed under 1.2 N force load using probe tip 7 mm in diameter for 250 cycles.


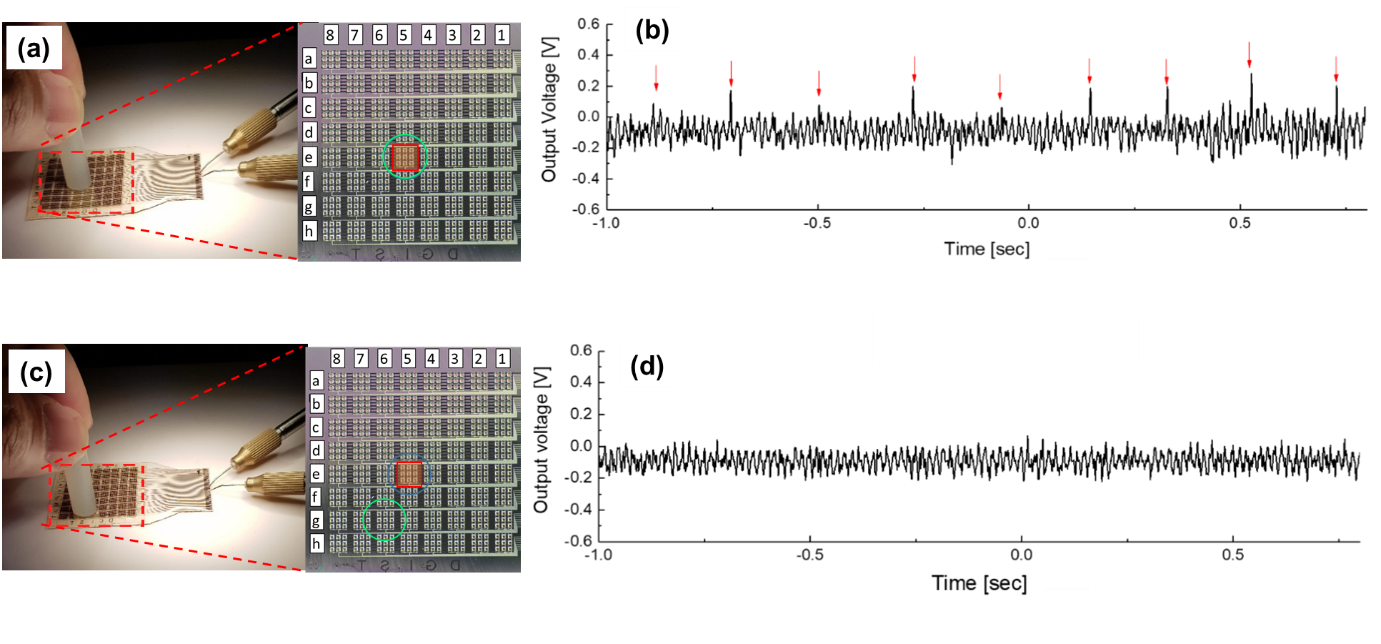


**Figure S6.** (a) Mechanical stimulation of the sensor and measurements in the activated channel. The red arrow indicates the stimulation signal under tapping with a rubber rod. (b) Measurement in the channel near the area (orange square) with applied mechanical stimulation area (green circle).
